# *Reineckia carnea* Alleviates the Production of Inflammatory Cytokines and MUC5AC in Rats with Chronic Obstructive Pulmonary Disease

**DOI:** 10.1155/2022/2135487

**Published:** 2022-06-16

**Authors:** Wei Liu, Jiang Li, Tongyao Li, Youliang Xie, Caihong Luo

**Affiliations:** ^1^Respiratory Medicine, The Second Affiliated Hospital of Guizhou University of Chinese Medicine, Guiyang, China; ^2^Prescription Teaching and Research Office, Guizhou University of Chinese Medicine, Guiyang, China; ^3^Respiratory Medicine, Qianxinan Hospital of Traditional Chinese Medicine, Guiyang, China; ^4^Department of Internal Medicine, Xiangxiang People's Hospital, Xiangtan, China; ^5^Geriatric Respiratory Department, Henan Electric Power Hospital, Zhengzhou, China

## Abstract

**Background:**

*Reineckia carnea* (RC), a perennial evergreen herb which belongs to *Reineckia* Kunth (Liliaceae), can be used for clearing the lungs and relieving cough, reducing phlegm and anti-inflammatory effects. Moreover, chronic obstructive pulmonary disease (COPD) is characterized by airway and lung inflammation and increased secretion of airway mucus. Therefore, RC has the potential to treat COPD.

**Methods:**

NR8383 cells were cultured and treated with various concentrations of RC (100 mg/mL, 10 mg/mL, 1 mg/mL, 100 *μ*g/mL, 10 *μ*g/mL, 1 *μ*g/mL, 100 ng/mL, and 10 ng/mL). Cell viability and levels of interleukin (IL)-1*β,* cyclooxygenase-2 (COX-2), and prostaglandin E2 (PGE2) in the cell culture supernatant or rat serum were analyzed using CCK-8 and enzyme-linked immunosorbent assay (ELISA), respectively. Sprague Dawley rats were assigned to mock, COPD model, RC (0.67 g/kg, 1.35 g/kg, and 2.7 g/kg), and ambroxol (5.4 mg/kg) groups. Western blot and quantitative polymerase chain reaction (qPCR) analyses were used to evaluate the protein and mRNA expression levels of mucin 5AC (MUC5AC) and Toll-like receptor 4 (TLR4).

**Results:**

The results showed that *Reineckia carnea* (RC) extract (RCE) inhibited the proliferation of NR8383 cells and suppressed the production of IL-1*β*, PGE2, and COX-2 in NR8383 cells. Moreover, RCE decreased the levels of IL-1*β,* PGE2, and COX-2 in the serum of rats with COPD and alleviated the expression of TLR4 and MUC5AC induced by COPD in rat lung tissue.

**Conclusion:**

RCE alleviated COPD by inhibiting the expression of COPD-induced inflammatory cytokines and MUC5AC in rats.

## 1. Introduction

Patients with chronic obstructive pulmonary disease (COPD) exhibit airway mucus hypersecretion with chest tightness, shortness of breath, fatigue aggravation, phlegm, and viscous mucus, resulting in airflow limitation. If not treated in a timely manner, progression to airway obstruction and asphyxia can occur [1, 2]. At present, COPD is often treated by inhaled bronchodilators and glucocorticoids, but there are many adverse reactions and the inhaled drugs are carried out by sputum due to the excessive secretion of airway mucus, which cannot exert their efficacy [3]. Therefore, the development of safe and effective treatments is urgently needed. Many studies have found that airway mucus hypersecretion is an important pathophysiological characteristic of COPD and an independent risk factor for COPD development and prognosis, and exists at all stages of disease occurrence and development [4, 5]. Although a study has demonstrated an important role of IL-1*β*/COX-2 in airway mucus hypersecretion [6], the molecular mechanism of airway mucus hypersecretion in COPD still needs to be elucidated.

Airway mucus hypersecretion in COPD is caused by a variety of factors, including inflammatory factors, oxidative stress, and multiple signaling pathways. In the IL-1*β/*COX-2 signaling pathway, interleukin-1*β,* cyclooxygenase-2 (COX-2), prostaglandin 2 (PGE2), and MUC5AC participate in the inflammatory reaction and lead to an increase in secretion from the glands, leading to airway mucus hypersecretion. Toll-like receptor (TLR) is a kind of innate immune receptor activated by the outer membrane lipopolysaccharide of Gram-negative bacteria and promotes the phosphorylation of a series of downstream factors [7]. TLR4 can regulate the inflammatory response. Studies have shown that TLR4 can activate the nuclear factor kappa-B (NF-*κ*B) signaling pathway and regulate the expression of inflammation-related factors. As the earliest inflammatory factor, IL-1*β* participates in the whole inflammatory process in COPD. Therefore, there may be a connection between TLR4 and IL-1*β*, leading to the inflammatory reaction and mucus hypersecretion.

*Reineckia carnea* (RC) is a Liliaceae plant, and the whole plant mainly contains various chemical components such as steroids, flavonoids, lignans, terpenes, and organic acids, which have pharmacological activities such as antihemolysis, relieving cough, reducing phlegm, anti-inflammatory, analgesic, and hypoglycemic effects, and killing snails [8, 9]. Therefore, RC is commonly used to treat various diseases, especially respiratory diseases, such as lung dryness and cough, and is used by the Miao group to treat bronchitis, pneumonia, and other diseases [10]. Extensive research at the Guiyang College of traditional Chinese medicine has shown that RCE can effectively dilute sputum and reduce sputum secretion in patients with COPD. Furthermore, modern pharmacological studies have shown that RCE relieves cough, resolves phlegm, reduces inflammation, reduces airway secretion, and inhibits airway mucosal hypersecretion in COPD [11]. In addition, a study showed that after being injected subcutaneously with a 4500 mg/kg injection made with RC, all 12 mice died within 7 days [12].

The above studies have shown that RC can be used to treat COPD. Therefore, in this study, we explored the molecular mechanism by which RCE inhibits inflammation in COPD, with a focus on the IL-1*β*/Cox-2 signaling pathway, via animal experiments in vivo and cells assay in vitro, which provides strong evidence for its clinical use in the treatment of COPD. Moreover, ambroxol hydrochloride is an expectorant commonly used for the treatment of COPD, with anti-inflammatory and antioxidant effects. Therefore, ambroxol hydrochloride was selected as a control in the analyses of the therapeutic effect of RCE.

## 2. Materials and Methods

### 2.1. Animals

Ninety healthy male Sprague Dawley (SD) rats without specific antigens were provided by Chongqing Tengxin Biotechnology Co., Ltd. Before the experiment, the 6- to 8-week-old rats, weighing 180–220 grams, acclimatized for a week in a clear plastic cage with free of specific pathogens, a constant 12-hour light-dark cycle, and free access to water and chow.

### 2.2. Establishment of COPD Rat Models

Sixty-seven rats were randomly selected to construct COPD rat models. COPD rat models were established in 12 weeks by intranasal instillation of lipopolysaccharide (LPS) and cigarette smoke inhalation, as previously described [8, 13, 14]. 200 *μ*L LPS (catalog number: ST1470, Beyotime, Shanghai, China) (30 *μ*g/6 *μ*L) was administered to SD rats via the nasal cavity on days 1, 29, and 57. The rats were exposed to cigarette (China Tobacco Guizhou Industrial, Co., Ltd) smoke in an in-house glass fumigation box (70 cm × 50 cm × 30 cm) for 30 min, 5 days per week for 12 weeks from days 2 to 84 (except on days 29 and 57). Subsequently, 12 COPD rats and 12 healthy rats were randomly selected to determine whether the COPD model was established successfully by HE staining of lung tissues and observing the lung pathological characteristics. The evaluation indices for successful model establishment were as follows: infiltration of inflammatory cells on the surface of the peripheral airway, bronchus, and bronchioles; hyperplasia and hypertrophy of the mucous glands and an increase in goblet cells; injury of the small airway, hyperplasia of collagen and scar tissue, and stenosis of the airway cavity; dilation and destruction of respiratory bronchioles, irregular expansion of alveoli, and formation of pulmonary bullae; hyperplasia of the smooth muscle of pulmonary arterioles; and infiltration of inflammatory cells in the vascular wall.

### 2.3. RCE Preparation

The RCE was prepared using the traditional method as previously described [8, 15]. In brief, 3000 g of dried RC was pulverized to a crude product and extracted with 15 times distilled water for 3 times, 2 h each time. The decoction was filtered and concentrated under pressure to obtain the extract which was dried and weighed. The extract was 645.36 g and the yield was 21.51%. Yield (%) = (weight of extract (*g*) ÷ weight of RC (*g*)) × 100%.

### 2.4. Animal Groups

The remaining COPD rats were randomly divided into 5 groups, COPD model, low-dose RC (LD-RC), medium-dose RC (MD-RC), high-dose RC (HD-RC), and ambroxol groups. Each group consisted of 11 rats, and the remaining 11 healthy rats were used as the negative control group named the mock group.

### 2.5. Treatment of Each Group of Animals

Based on the amount of medicine used per unit body surface area, the amount of medicine used in rats is about 6.3 times that of humans [8]. The daily dosage of RC for adults is 15–30 g, so the dosage for rats is 1.35–2.7 g/kg. The low-, medium-, and high-dose RC were 0.67 g/kg, 1.35 g/kg, and 2.7 g/kg, respectively, which converted to RCE were 0.14 g/kg, 0.29 g/kg, and 0.58 g/kg, respectively. The daily dosage of ambroxol (Shanxi C&Y Pharmaceutical Group Co., Ltd) for adults is 60 mg, so the dosage for rats is 5.4 mg/kg. The drugs were separately dissolved in 3 mL of saline and gavaged to the corresponding groups of rats once a day for 17 days.

In the mock and COPD model groups, normal saline (3 mL/rat) was delivered by gavage once a day for 17 days. The experiments of this study were completed according to the requirements of the Animal Experiment Ethics Committee of the Guizhou University of Traditional Chinese Medicine.

### 2.6. Cell Culture

Rat alveolar macrophages and NR8383 cells (Xiamen Immocell Biotechnology Co., Ltd, catalog number: IM-R006) were cultured in an RPMI 1640 medium (Gibco, New York, USA) containing 10% fetal bovine serum (FBS, Gibco) at 37°C and 5% CO_2_. NR8383 cells were cultured in suspension, and the solution was changed in 2-3 days.

### 2.7. CCK-8 Assay

NR8383 cells were cultured overnight in 96-well plates at a density of 10000 cells per well. The cells were treated with different concentrations of RCE (1, 10, and 100 mg/mL; 1, 10, and 100 *μ*g/mL; 10 and 100 ng/mL) or ambroxol (MedChemExpress, catalog number: HY-B1039) (10, 20, and 30 *μ*g/mL). After 24 h, the cells were detected using CCK-8 (MedChemExpress, catalog number: HY-K0301, Shanghai, China) and a microplate reader (Bio Tek) according to the manufacturer's instructions. Results are presented as the mean ± standard deviation (SD) of six wells.

### 2.8. Cell Immunohistochemistry

The NR8383 cells were treated with 100 mg/mL RC or 30 *μ*g/mL ambroxol. After 24 h, the cells were fixed on the slides with 0.4% paraformaldehyde, which was incubated successively with 0.5% Triton X-100 (Thermo Fisher Scientific, catalog number: R21902) for 20 min and then with 3% H_2_O_2_ for 15 min. After being incubated with 5% FBS for 30 min, the cells were incubated with the TRL4 antibody (Abcam, catalog number: ab22048, dilution rate: 1 : 100) or MUC5AC antibody (Abcam, catalog number: ab3649, dilution rate: 1 : 100) for 2 h, followed by HRP-conjugated goat anti-mouse IgG (Abcam, catalog number: ab205719, dilution rate: 1 : 1000) for 1 h. Subsequently, the cells were stained with a DAB Immunohistochemistry Color Development Kit (Sangon Biotech, catalog number: E670033) and the nuclei were stained with hematoxylin. The cells were viewed under a microscope and photographed. The experiments were performed thrice independently.

### 2.9. Enzyme-Linked Immunosorbent Assay (ELISA)

The levels of IL-1*β*, PGE2, and COX-2 in the cell culture supernatant or serum were determined by Rat IL-1 beta ELISA kit (Abcam, catalog number: ab255730), Rat PGE2 ELISA Kit (Wuhan Fine Biotech Co., Ltd, catalog number: ER1800), ELISA kits, and Rat COX-2 ELISA Kit (CUSABIO TECHNOLOGY, catalog number: CSB-E13399r) according to the manufacturer's instructions. The experiments were performed more than three times independently.

### 2.10. Isolation of RNA and Quantitative PCR (qPCR)

RNA was isolated from the lung tissues of rats using an RNA reagent (Takara, Kusatsu, Japan) and we used a PrimeScript™ RT Master Mix (Takara, catalog number: RR036Q) to reverse transcribe into cDNA. And then we employed the obtained cDNA, TB Green® Fast qPCR Mix (Takara, catalog number: RR430S), the Bio-Rad iQ5 Real-Time PCR System, and primers which are shown in Table 1, to perform qPCR. The data are expressed as the mean ± SD of three independent experiments.

### 2.11. Western Blot Analysis

Western blotting was used to examine the expression of MUC5AC and TLR4. In order to extract proteins, we added RIPA buffer (Beyotime, Shanghai, China) to the ground tissue and added protease inhibitors (Roche, Basel, Switzerland). To measure the protein concentrations, we used the BCA Protein Analysis Kit (Pierce, Rockford, IL, USA) according to the manufacturer's instructions. Thereafter, we put 50 *μ*g of protein lysate in each SDS-PAGE well to separate proteins and transferred the proteins to a PVDF membrane which was then sealed with 5% milk at room temperature for 1 hour, followed by overnight incubation with TRL4 antibody (Abcam, catalog number: ab217274, dilution rate: 1 : 300), MUC5AC antibody (Abcam, catalog number: ab3649, dilution rate: 1 : 1000), or *β*-actin antibody (Abcam, catalog number: ab6276, dilution rate: 1 : 5000), at 4°C. After the membrane was washed with TBST, we incubated the membrane with HRP-conjugated goat anti-rabbit IgG (Abcam, catalog number: ab205718, dilution rate: 1 : 10000), or HRP-conjugated goat anti-mouse IgG (Abcam, catalog number: ab205719, dilution rate: 1 : 10000) for 1 hour at room temperature. The Pierce™ ECL Western Blotting Substrate (Thermo Scientific, catalog number: 32109) was used for luminescent detection. A gel imaging system was used to collect images, and Image Lab 3 was used to analyze gray values. Protein levels were normalized against *β*-actin. The experiments were performed thrice independently.

### 2.12. Statistical Analysis

We used the SPSS 20.0 software for statistical analyses. To compare the differences between two groups and between multiple groups, we performed Student's *t*-test (unpaired) and analysis of variance (ANOVA), respectively. A value of *P* < 0.05 was regarded as statistically significant.

## 3. Results

### 3.1. RCE Inhibits the Production of IL-I*β,* PGE2, and COX-2 in NR8383 Cells

**Figure 1:**
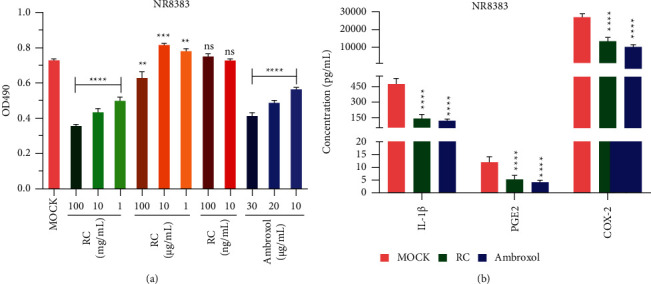
RCE inhibits the production of IL-I*β,* PGE2, and COX-2 in NR8383 cells. (a) The effects of RC and ambroxol on NR8383 cell proliferation. (b) After the NR8383 cells were treated with 100 mg/mL RC or 30 *μ*g/mL ambroxol for 24 h, the effects of RCE and ambroxol on the production of IL-I*β*, PGE2, and COX-2 in NR8383 cells. RC : *Reineckia carnea,* ns: not significant, ^*∗∗*^*P* < 0.01, ^*∗∗∗*^*P* < 0.001, ^*∗∗∗∗*^*P* < 0.0001.

**Figure 2:**
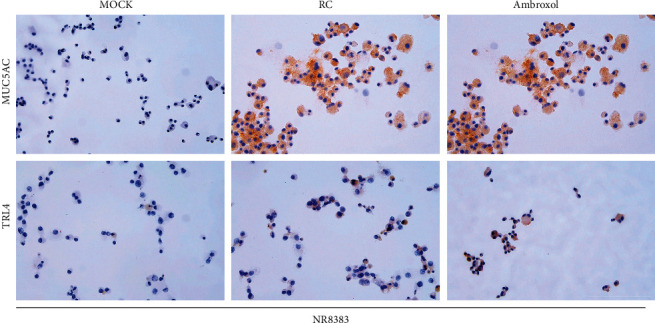
Immunohistochemical representative images show the expression of MUC5AC and TLR4 in RC- or ambroxol-treated NR8383 cells. RC : *Reineckia carnea.*

We investigated the effects of various concentrations of RC on the proliferation of NR8383 cells. For RC concentrations of 1 mg/mL, 10 mg/mL, and 100 mg/mL, cell proliferation gradually decreased in a concentration-dependent manner (Figure 1(a), *P* < 0.05). Compared with cells not treated with RC (mock group), there was no significant difference in cell proliferation after the cells were treated with 100 ng/mL and 10 ng/mL RC (Figure 1(a), *P* < 0.05). As the ambroxol concentration increased, cell proliferation decreased in a concentration-dependent manner (Figure 1(a), *P* < 0.05). Based on these results, an RC concentration of 100 mg/mL and ambroxol concentration of 30 *μ*g/mL were selected for further analyses, and the levels of IL-I*β,* PGE2, and COX-2 in the NR8383 culture medium were analyzed. RCE and ambroxol inhibited the production of IL-I*β,* PGE2, and COX-2 (Figure 1(b), *P* < 0.01). The expression of MUC5AC and TLR4 in NR8383 cells was evaluated by immunohistochemistry. Compared with the mock group, MUC5AC and TLR4 levels in the RC and ambroxol groups were significantly (*P* < 0.01) lower (Figure 2).

### 3.2. RCE Decreases the Levels of IL-I*β,* PGE2, and COX-2 in Serum of COPD Model

**Figure 3:**
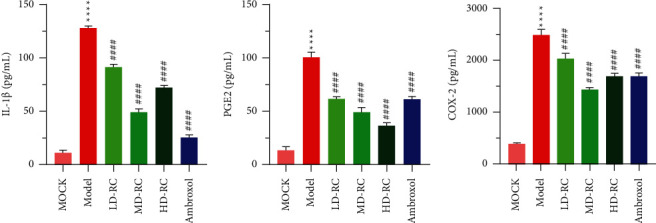
The ELISA results of the serum levels of IL-I*β,* PGE2, and COX-2. LD-RC : The rats were given a low dose of *Reineckia carnea* extract (0.14 g/kg) dissolved in 3 mL of saline. MD-RC : The rats were given a medium dose of *Reineckia carnea* extract (0.29 g/kg) dissolved in 3 mL of saline. HD-RC : The rats were given a high dose of *Reineckia carnea* extract (0.58 g/kg) dissolved in 3 mL of saline. ^*∗∗∗∗*^*P* < 0.0001 vs. mock; ^####^*P* < 0.0001 vs. Model.

To investigate the molecular mechanism of RCE treatment of COPD, we constructed a COPD rat model. After the models were treated with RCE or ambroxol, the serum was collected to detect the levels of IL-I*β,* PGE2, and COX-2. As shown in Figure 3, compared with the mock group, the levels of IL-1*β,* PGE2, and COX-2 in the model group were significantly higher (Figure 3, *P* < 0.01). Compared with the model group, the levels of IL-1*β*, PGE2, and COX-2 were lower in the RC and ambroxol groups (Figure 3, *P* < 0.01). These results indicate that RCE has similar effects as ambroxol in reducing inflammatory indices in rats with COPD.

### 3.3. RCE Downregulates the COPD-Induced Expression of MUC5AC and TLR4 in Rat Lung Tissues

**Figure 4:**
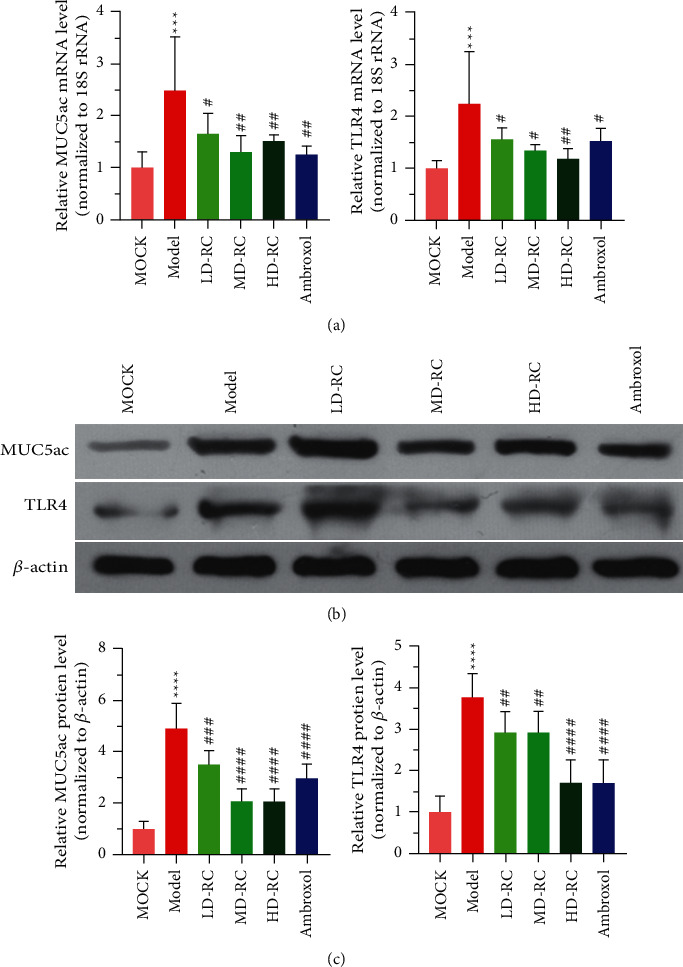
RCE downregulates the COPD-induced expression of MUC5AC and TLR4 in rat lung tissues. (a) The expression levels of MUC5AC and TLR4 in different groups. (b) The results of western blot analysis of MUC5AC and TLR4 levels. (c) Histogram of relative expression levels of TLR4 and MUC5AC proteins. LD-RC : The rats were given a low dose of *Reineckia carnea* extract (0.14 g/kg) dissolved in 3 mL of saline. MD-RC : The rats were given a medium dose of *Reineckia carnea* extract (0.29 g/kg) dissolved in 3 mL of saline. HD-RC : The rats were given a high dose of *Reineckia carnea* extract (0.58 g/kg) dissolved in 3 mL of saline. *∗∗∗P* < 0.001, *∗∗∗∗P* < 0.0001 vs. mock; #*P* < 0.05, ##*P* < 0.01, ###*P* < 0.001, and ####*P* < 0.0001 vs. Model.

Next, the expression levels of *MUC5AC* and *TLR4* in different groups were analyzed. The mRNA levels of *MUC5AC* and *TLR4* in the model group were significantly higher than those in the mock group (Figure 4(a), *P* < 0.01). After the rats with COPD were treated with RCE or ambroxol, the mRNA levels of *MUC5AC* and *TLR4* were significantly decreased (Figure 4(a), *P* < 0.05).

Western blot analysis showed that MUC5AC and TLR4 levels in the model group were significantly higher than those in the mock group (Figures 4(b) and 4(c), *P* < 0.01). The levels of TLR4 and MUC5AC proteins in the lung tissues of COPD rats treated with RCE or Ambroxol were significantly lower than those in the model group (Figures 4(b) and 4(c), *P* < 0.01). Together, RCE attenuated COPD-induced TLR4 and MUC5AC expression.

## 4. Discussion

COPD, an inflammatory disease in the lung parenchyma and respiratory tract, is caused by continued inhalation of toxic substances, predominantly cigarette smoke [4, 16]. Moreover, LPS can cause inflammation [17]. Therefore, we used cigarette smoke and LPS to generate a rat COPD model. Furthermore, the IL-1*β*/COX-2 signaling pathway can cause airway mucus hypersecretion in COPD [18]. Inflammatory factors bind to corresponding receptors to activate COX-2, leading to increased secretion [19]. Airway mucus hypersecretion in COPD is related to an inflammatory reaction [20]. COX-2 is transformed into prostaglandin products *in vivo*. After the transformation, prostaglandin E2 is activated, increasing vascular permeability and dilating blood vessels, causing mucosal edema, increasing gland secretion, and affecting airway ventilation and ventilation function [21]. In this study, we evaluated the effect of RCE on inflammatory response in rat alveolar macrophages *in vitro* and *in vivo.* RCE and ambroxol had similar effects; they reduced the levels of IL-1*β*, PGE2, and COX-2 to relieve COPD.

In addition, COX-2 is an inducible enzyme that is rapidly synthesized when cells are stimulated by various factors. It can be induced in bronchial epithelial cells and alveolar epithelial cells. Macrophages in alveoli are the main inflammatory cells expressing COX-2. When IL-1*β* binds to its receptor, the activity of COX-2 is increased. COX-2 has a catalytic role in the conversion of arachidonic acid to PGE and PGE2 under the action of PGE synthetase [22]. PGE2 can increase vascular permeability and dilate blood vessels, resulting in bronchial mucosal edema, increased gland secretion, and airway obstruction by exudates [23]. The expression of MUC5AC and TLR4 in rat alveolar macrophages was detected by immunohistochemistry. Compared with the mock group, MUC5AC and TLR4 levels in the RC and ambroxol groups were significantly lower. IL-1*β* contributes to the early inflammatory response and can be secreted by a variety of cells, including epithelial cells, macrophages, and neutrophils. Lappalainen et al. used a transgenic animal model to show that IL-1*β* can cause alveolar septum enlargement, tracheal wall thickening, and increased mucus secretion, consistent with the pathological changes in COPD. IL-1*β* can activate macrophages and other inflammatory cells to release inflammatory mediators, which can cause pulmonary inflammation, characterized by macrophage and granulocyte infiltration. IL-1*β* may be a cause of chronic lung inflammation.

RCE relieves cough, resolves phlegm, and has anti-inflammatory effects [24]. It is often used to treat cough, bronchitis, pneumonia, and other diseases by the Miao group in China. RCE-4 from RC, which suppresses the PI3K/Akt/mTOR signaling pathway and NF-*κ*B activation, and has an antiproliferative effect on HeLa cells, so RC mainly reduces inflammation and oxidative stress [1].

Studies have shown that RCE can improve lung function and reduce sputum secretion in patients with COPD; however, few studies have examined the molecular mechanisms underlying its effects. ELISA was used to analyze the effects of various concentrations of RC on the serum levels of IL-1*β*, PGE2, and COX-2 in a rat model of COPD. Levels of IL-1*β*, PGE2, and COX-2 were high in the mock group. RCE and ambroxol could inhibit the secretion of airway mucus in rats with COPD. For some concentrations, the effects of RCE were more potent than those of ambroxol.

TLR is an innate immune receptor involved in the regulation of the inflammatory response. In COPD, the inflammatory response activates the IL-1*β*/COX-2 signaling pathway, leading to MUC5AC overexpression and airway mucus hypersecretion. Various factors activate the TLR4 receptor, which regulates the expression of inflammatory factors and causes inflammatory reactions via various enzymes. The overexpression of IL-1*β* can increase the activity of COX-2, increase the synthesis of PGE2, increase blood permeability and vasodilation, cause bronchial mucosal edema, increase secretion from glands, and lead to airway restriction. Based on analyses of protein and mRNA levels, MUC5AC and TLR4 were upregulated in the mock group, indicating that mucus hypersecretion in COPD is related to the high expression of these loci. MUC5AC and TLR4 were inhibited by both RC and ambroxol (*P* < 0.01).

The expression levels of MUC5AC and TLR4 were analyzed by PCR and western blotting analyses. As the RC concentration increased, the mRNA and protein expression levels of MUC5AC and TLR4 in rats with COPD decreased significantly, and a similar effect was observed in the ambroxol group. The results showed that ambroxol could inhibit airway mucus hypersecretion in COPD; however, medium and high doses of RC were more effective than those of ambroxol. Lu et al. confirmed that sequential treatment with Tongsai and Bufei Yishen granules during acute exacerbations of chronic obstructive pulmonary disease risk window (AECOPD-RW) periods reduces the inflammatory response, improves pulmonary function, and shortens the recovery course, particularly when Chinese and Western medicines are integrated [25]. In a recent study, it was shown that Bufei Yishen granules combined with electroacupuncture was effective in the treatment of COPD in rats, and the combination is superior to either strategy alone [26]. Lin et al. have shown that curcumin inhibits LPS-induced airway mucus hypersecretion and inflammation which are important pathophysiological features of chronic inflammatory airway diseases [27].

The study was based on rats and rat cells, and the results cannot be directly transferable to humans, which is a limitation of the study.

## 5. Conclusion

In conclusion, our results revealed that RCE inhibited the proliferation of NR8383 cells, and suppressed the production of IL-1*β*, PGE2, and COX-2 in NR8383 cells. Moreover, RCE alleviated the expression of IL-1*β*, PGE2, COX-2, TLR4, and MUC5AC induced by COPD. This study preliminarily revealed the molecular mechanism of RCE treatment of COPD to provide a theoretical basis for the treatment of COPD by RC and for better exploitation and quality control of RC.

## Figures and Tables

**Table 1:** Oligonucleotide primers targeting rat genes for qPCR.

Gene	Forward primer sequence (5'–3')	Reverse primer sequence (5'–3')
MUC5AC	AATGGCTACCTGAAGGTGGTGG	AAACTCGCTGGATTCTGGACTG
TLR4	AACATGAGTCACAACAACCTAC	TATTCACATATACAAGCAACAG
*β*-Actin	TCTGTGATGCCCTTAGATGTCG	AATGGGGTTCAACGGGTTAC

## Data Availability

The datasets used and analyzed during the current study are available from the corresponding author on reasonable request.

## References

[1] Bai C., Yang X., Zou K. (2016). Anti-proliferative effect of RCE-4 from *Reineckia carnea* on human cervical cancer HeLa cells by inhibiting the PI3K/Akt/mTOR signaling pathway and NF-*κ*B activation. *Naunyn-Schmiedeberg’s Archives of Pharmacology*.

[2] Tanner L., Single A. B. (2020). Animal models reflecting chronic obstructive pulmonary disease and related respiratory disorders: translating pre-clinical data into clinical relevance. *Journal of Innate Immunity*.

[3] Chronic Obstructive Pulmonary Disease Committee RS (2021). Guidelines for the diagnosis and management of chronic obstructive pulmonary disease revised version 2021. *Chinese Journal of Tuberculosis and Respiratory Diseases*.

[4] Akata K., van Eeden S. F. (2020). Lung macrophage functional properties in chronic obstructive pulmonary disease. *International Journal of Molecular Sciences*.

[5] Samsuzzaman M., Uddin M. S., Shah M. A., Mathew B. (2019). Natural inhibitors on airway mucin: molecular insight into the therapeutic potential targeting MUC5AC expression and production. *Life Sciences*.

[6] Zhou L., Liu Y., Chen X. (2018). Over-expression of nuclear factor-& kappa; B family genes and inflammatory molecules is related to chronic obstructive pulmonary disease. *International Journal of Chronic Obstructive Pulmonary Disease*.

[7] Li J., Ye Z. (2020). The potential role and regulatory mechanisms of muc5ac in chronic obstructive pulmonary disease. *Molecules (Basel, Switzerland)*.

[8] Chen J. B., Zhang L. J., Qiu N. N. (2020). Study on fluid extract from herba euphorbiae in the treatment of chronic obstructive pulmonary disease and its mechanism. *Journal of Guizhou Medical University*.

[9] Liu H., Yang J. Q., Xiong L., Wang Y. (2012). Study on chemical constituents and pharmacological activities of *Reineckia carnea*. *Chinese Traditional Patent Medicine*.

[10] Han N., Chang C., Wang Y., Huang T., Liu Z., Yin J. (2010). Th*e in vivo* expectorant and antitussive activity of extract and fractions from *Reineckia carnea*. *Journal of Ethnopharmacology*.

[11] Zhang Y., Du J., Xu J., Yao G., Wang L., Zhang L. (2006). Pharmacological study of total Saponins from Reineckia carnea on hemolysis, relieving a cough, abolishing phlegm and anti -inflammation. *Medical Journal of the Chinese People’s Armed Police Forces*.

[12] Qiu D. W., Du J. (2008). *GUIZHOU SHIDA MIAOYAO YANJIU: Traditional Chinese Medicine*.

[13] Jia M., Fan W. H., Qin Q. H., Zhang H., Chen Y. L. (2019). Cigarette dust particles induced lung function injury of chronic obstructive pulmonary disease in mice. *Chinese Journal of Pathophysiology*.

[14] Cheng Y., Tao W. L., Zhang X. M., Yang Z. S., Chen J. (2016). Experimental study on the building animal model of chronic obstructive pulmonary disease. *World Journal of Integrated Traditional and Western Medicine*.

[15] Xu X., Wu B., Li Y. (2018). Isolation and identification of chemical constituents from Miao medicine reineckia carnea in Guizhou. *Chinese Journal of Experimental Traditional Medical Formulae*.

[16] Wang C., Zhou J., Wang J. (2020). Progress in the mechanism and targeted drug therapy for COPD. *Signal Transduction and Targeted Therapy*.

[17] Ren Q., Zhao S., Ren C., Ma Z. (2018). Retracted: astragalus polysaccharide alleviates LPS-induced inflammation injury by regulating miR-127 in H9c2 cardiomyoblasts. *International Journal of Immunopathology and Pharmacology*.

[18] Tang F., Ling C. (2019). Curcumin ameliorates chronic obstructive pulmonary disease by modulating autophagy and endoplasmic reticulum stress through regulation of SIRT1 in a rat model. *Journal of International Medical Research*.

[19] Chen B., You W.-J., Xue S. (2016). Overexpression of farnesoid X receptor in small airways contributes to epithelial to mesenchymal transition and COX-2 expression in chronic obstructive pulmonary disease. *Journal of Thoracic Disease*.

[20] Wang X., Yang X., Li Y. (2017). Lyn kinase represses mucus hypersecretion by regulating IL-13-induced endoplasmic reticulum stress in asthma. *EBioMedicine*.

[21] Zago M., Sheridan J. A., Traboulsi H. (2017). Low levels of the AhR in chronic obstructive pulmonary disease (COPD)-derived lung cells increases COX-2 protein by altering mRNA stability. *PLoS One*.

[22] Puratchikody A., Umamaheswari A., Irfan N. (2019). A novel class of tyrosine derivatives as dual 5-LOX and COX-2/mPGES1 inhibitors with PGE2 mediated anticancer properties. *New Journal of Chemistry*.

[23] Ho M.-Y., Liang S.-M., Hung S.-W., Liang C.-M. (2019). Retraction: MIG-7 controls COX-2/PGE2-mediated lung cancer metastasis. *Cancer Research*.

[24] Zheng L., Zhang D., Li Y. (2019). Three new steroidal components from the roots of *Reineckia carnea*. *Natural Product Research*.

[25] Lu X., Li Y., Li J. (2016). Sequential treatments with Tongsai and Bufei yishen granules reduce inflammation and improve pulmonary function in acute exacerbation-risk window of chronic obstructive pulmonary disease in rats. *Evidence-Based Complementary and Alternative medicine*.

[26] Ma J., Tian Y., Li J. (2019). Effect of Bufei Yishen granules combined with electroacupuncture in rats with chronic obstructive pulmonary disease via the regulation of TLR-4/NF-*κ*B signaling. *Evidence-Based Complementary and Alternative Medicine*.

[27] Lin X.-P., Xue C., Zhang J.-M., Wu W.-J., Chen X.-Y., Zeng Y.-M. (2018). Curcumin inhibits lipopolysaccharide-induced mucin 5AC hypersecretion and airway inflammation via nuclear factor erythroid 2-related factor 2. *Chinese Medical Journal*.

